# Robotic Seals as Therapeutic Tools in an Aged Care Facility: A Qualitative Study

**DOI:** 10.1155/2016/8569602

**Published:** 2016-11-20

**Authors:** Melanie Birks, Marie Bodak, Joanna Barlas, June Harwood, Mary Pether

**Affiliations:** ^1^Nursing, Midwifery, and Nutrition, Centre for Nursing and Midwifery Research, College of Healthcare Sciences, James Cook University, Townsville, QLD, Australia; ^2^Nursing, Midwifery, and Nutrition, College of Healthcare Sciences, James Cook University, Townsville, QLD, Australia; ^3^Clinical Psychology, College of Healthcare Sciences, James Cook University, Singapore Campus, Singapore; ^4^Aged Care Services Australia Group, Launceston, TAS, Australia; ^5^Regis Aged Care, Townsville, QLD, Australia

## Abstract

Robots, including robotic seals, have been used as an alternative to therapies such as animal assisted therapy in the promotion of health and social wellbeing of older people in aged care facilities. There is limited research available that evaluates the effectiveness of robot therapies in these settings. The aim of this study was to identify, explore, and describe the impact of the use of Paro robotic seals in an aged care facility in a regional Australian city. A qualitative, descriptive, exploratory design was employed. Data were gathered through interviews with the three recreational therapists employed at the facility who were also asked to maintain logs of their interactions with the Paro and residents. Data were transcribed and thematically analysed. Three major themes were identified from the analyses of these data: “a therapeutic tool that's not for everybody,” “every interaction is powerful,” and “keeping the momentum.” Findings support the use of Paro as a therapeutic tool, revealing improvement in emotional state, reduction of challenging behaviours, and improvement in social interactions of residents. The potential benefits justify the investment in Paro, with clear evidence that these tools can have a positive impact that warrants further exploration.

## 1. Introduction

Residential aged care facilities provide an important service in promoting the health and social wellbeing of elderly individuals. Over 230,000 individuals currently reside in aged care facilities in Australia, with approximately 52% having a diagnosis of dementia [1]. Recreational therapy is a significant component of the health care regime for aged care residents and is effective in promoting the physical, social, and psychological wellbeing of all older adults residing in aged care facilities.

Animal assisted therapy is perhaps the best known form of recreational therapy in the aged care setting. Animal assisted therapy began in the USA in the 1960s and, despite the recognised benefits of this form of therapy for use with the elderly, there is minimal research on its effectiveness [2]. The limited amount of recent work that has been done in this area emanates from Italy. These studies suggest that animal assisted therapy reduces depressive symptoms and agitation and results in an overall positive experience for the resident [2–4]. Animal assisted therapy is not without its problems, however, which has seen these programs fall out of favour because of limited availability of appropriate animals and issues of hygiene and safety [5]. As a result, robots, and particularly pet robots, have arisen as a viable alternative.

Much of the recent research in the area of robots in healthcare has arisen from Canada [6–8], New Zealand [9–11], Europe [12–16], and Australia [17]. While some of this work explores robots generally [7, 10], most examines the use of robots with the elderly in broad terms [9, 13, 14, 16, 17], with some focus on dementia [6, 11, 12, 15] and disability [8].

A number of systematic [12, 17] and other structured [6, 13] reviews of the literature form much of the current work. These reviews raise issues in respect of worth of earlier research, arguing for greater emphasis on rigour and overall quality. Some studies, for example, simply compare one type of robot or system with another [10, 11], with limited consideration for the absolute value of robots in a given setting. In another study, Wolbring and Yumakulov [8] delivered an online survey, with no ability of the respondents to actually view the capabilities of the robot.

The Paro robotic seal is a socially responsive robot that has been in use since 2003 [18]. This high technology therapeutic tool reacts to the individual in response to the way in which it is treated. Paro has been shown to effect connections with elderly individuals who may be socially, cognitively, or emotionally isolated [19]. Research into seal robots provides an example of the conflict of interests evident in this field of research. Takanori Shibata, the inventor of the Paro robotic seal, has undertaken various studies into the use and value of his product. One example is the administration of a questionnaire sent with the robot when purchased [20], with a subsequent study comparing video recordings of elderly persons with dementia interacting with Paro versus a stuffed toy lion [21].

Other independent researches support the value of Paro. One group of researchers in the USA [22, 23] used observations and video recordings of participants interacting with Paro [23] or combined these with interviews with therapists to identify the positive potential that Paro has when used with people living with dementia [22]. Researchers from New Zealand [24] similarly used video and interviews with a broad range of residents and staff at an aged care facility to identify primarily social benefits. Ahn et al. [9], also from New Zealand, used interview, observation, and nonrandomised and randomised trials to add physical benefits to the list of positive outcomes. Studies from Europe mostly relied on quantitative methods [25, 26] to further demonstrate the potential value of Paro in dementia care in particular.

Most of the existing literature examining the effects of Paro describes studies conducted with residents of care facilities in almost all cases focusing on the care of persons with dementia. The research presented in this paper aims to contribute to the existing body of knowledge by exploring the experiences of therapists using Paro as a therapeutic tool with a more diverse group of residents in an aged care facility in regional Australia.

## 2. Materials and Methods

This study employed a qualitative descriptive, exploratory design. The setting was an aged care facility in a regional Australian city. This 127-bed facility was operated by a Nongovernment Organisation (NGO) and employed 125–130 staff. The research team was contacted by the facility when the robotic seals were purchased with an invitation to assist with research to evaluate their effectiveness. The research team attended an event to launch the use of the robotic seals (named “George” and “Sally” by the residents) in the facility and at this time explained the purpose of the research and the study design to the staff, residents, family, and friends in attendance. The original intention was to recruit from this broader pool of stakeholders. The three recreational (diversional) therapists employed at the facility were those most involved with the use of the robotic seals, however, and it was these staff who participated in the study.

Prior to implementing the robotic seals project, the therapists participated in a number of short training sessions (tool boxes) led by the facility manager, who had worked previously with the Paro robotic seals. These workshops aimed to develop foundational understanding of how these robotic seals could be used as a therapeutic tool to benefit the day-to-day experiences of the residents. Following approval by the university's Human Research Ethics Committee, the therapists were asked to maintain a journal of their interactions with residents when using Paro. Paro was employed daily as a diversional therapy with selected residents in either an individual or group activity. Each session was 30–40 minutes in duration. During these sessions, residents were encouraged to interactively engage with Paro including stroking, cuddling, and speaking to the robotic seal.

After the seals had been in use for a period of approximately four months, semistructured interviews were conducted with the participants for approximately one hour each. While a group interview was planned, one therapist was unavailable and so was interviewed separately from the other two. Two members of the research team undertook an inductive thematic analysis of the data obtained from the verbatim interview transcripts and therapists' journals. This analysis was checked by a third researcher and the results are presented in Section 3.

## 3. Results

The findings of this study present an overview of the impact of Paro robotic seals on residents, from the perspective of the recreational therapists. The following discussion present an integrated analysis of the interviews and participant journals. The major themes derived from the analysis were as follows:* a therapeutic tool that's not for everybody*;* every interaction is powerful*; and* keeping the momentum*.

### 3.1. A Therapeutic Tool That's Not for Everybody

This theme encompasses the diversity of reactions to the Paro seals. In the case of residents, participants noticed a range of initial reactions from outright dismissal through curiosity to excitement. Those residents who dismissed the robots were noted by the participants to be more negative in their use of language. Amongst those who were curious, some apprehension was observed, which was overcome with encouragement from the participants.

*There have been a couple of residents that have been a little bit wary of George and Sally. I've gone back to those residents, and some of them have actually come forward and stroked George or Sally. The others have dismissed me straight away; don't want to have anything to do with it. [Participant 1]*

*I believe over 50 per cent are really interested to know more about this and then we get those who think it's a load of rubbish. [Participant 3]*

These varying initial reactions were thought to be driven by normal individual differences and differences relating to mental and physical health status. The participants in this study acknowledged and respected individual preferences for one activity over another. There was a sense that these healthcare workers viewed the robots as another optional activity that could form part of a therapeutic toolbox to draw on when working with elderly residents.

*…it is a therapeutic tool, but still I don't think it's for everybody. [Participant 1]*

Participants observed that Paro elicited more of a response from residents with dementia, even those with more advanced-stage dementia. There was a suggestion that some residents with dementia appeared to lack understanding that it was not real.

*I think the [residents with] dementia react better. They smile a lot more, or they frown, or their eyes sparkle. [Participant 1]*

*I think the interaction particularly with somebody with dementia who's like third stage was just wonderful. [Participant 3]*

In contrast, those who were more immediately dismissive of Paro were described by the participants as more cognitively able or* “mentally with-it.”* Differing reactions not only were dependent on whether or not the resident had dementia but seemed to relate to physical, cognitive, and emotional impairment more widely. According to the participants, those with depression and disabilities or those under palliative care, for example, were observed to respond positively to Paro.

*…one lady that there's nothing wrong [with cognitively], but physically she can't move or do anything. And her eyes just sparkle when I walk in and I put George or Sally on the bed table, because she can touch and stroke [him] and all that. [Participant 1]*

A diversity of reactions from residents' caregivers (family members and staff) to the Paro seals was also described. Participants in this study commented that the response from family members was primarily positive; they expressed interest in understanding the purpose of the Paros and shared views about the anticipated reactions from their relatives.

*…they've actually asked questions about where they came from and why we have them here, and most families have been very positive. [Participant 1]*

One of the participants observed the Paro facilitates an expression of affection between a resident who could not speak and her husband.

*…you could see the look on her face and his face and the touching which would - she touched his hand and they both touched Paro so it was a really wonderful experience. [Participant 3]*

Some negativity was observed, however, such as when derogatory comments were made about staff members using Paro:

*…somebody said to me, a family member said “oh, I've seen somebody carrying that around, they're like a complete idiot”. [Participant 3]*

The participants also observed staff expressing negative views about Paro. These included apparent concerns about the use of Paro infantilizing the residents by dismissing Paro as* “that toy.”* Negative reactions from other caregivers appeared to be driven by lack of understanding about the use of Paro. Some staff believed it was* “a waste of money*” and the participants in this study thought that these staff did not* “realize its benefits to the residents.” *One participant also reported that some staff expressed seemingly paranoid views about Paro being used as a recording device, perhaps with the intention of monitoring their behaviour at work.

*They walk away and the hands up “I'm not talking - I'm not talking while that's around and while it's turned off”. Then you're in the nurses' station, “is that turned off?” [Participant 3]*

### 3.2. Every Interaction Is Powerful

A number of therapeutic benefits for residents were observed by participants. Three subthemes were developed in this category. These subthemes encapsulated the emotional, behavioural, and social benefits of the Paro seals.

One common theme related to the emotional benefits. In one example, a participant reported that during a 15- to 20-minute one-on-one session some residents* “came out of their shells”* and seemingly had more* “spark”* in them.

*On entering the room I noticed her looking forlorn, distant and looking into space… she spotted George (Paro) in my arms and her whole body language changed immediately by showing exuberance, verve in her movements and in her face. She immediately held out her arms to hold George. [Participant 3 log entry]*

In some cases, participants reported that they witnessed an emotional bond developed between the resident and the robot. The Paro became something towards which they could express affection, talk, and welcome back like an old friend.

*He invited me into his room. As soon as he viewed George his eyes opened wide and a huge smile appeared on his face. With his focus on George, he placed his water on the bed side table and opened out his arms ready to take hold of him and commenced talking to George as if he was continuing his conversation from the last visit with Sally. He commenced with, “What have you been doing with yourself? “ Conversation flowed as he held George. [Participant 3 log entry]*

Participants also suggested that the emotional benefits extended to providing happiness and comfort at the end of life. This phenomenon was just one example of the powerful effect that these simple therapeutic tools can have.

*I used it on a palliative care resident and that was - it was a wonderful experience because she was able to verbalise how she was feeling [through] the tactile - feeling, the sensory part… “Yes dear, yes, I am feeling…” she looked at Paro “yes dear, I'm feeling like this”… I think that…she could see that she was thinking about her thoughts and she wanted to pass it on to somebody. [Participant 3]*

*George came in, he jumped - generated conversation. Even only a small bit of conversation but at least he was able to verbalise. I think in the last legs of his life, he was happy. [Participant 3]*

Behavioural benefits followed as a result of interacting with the robotic seals. The participants in this study were very excited by the positive influence the interaction with the Paro was having for the residents. They used adjectives such as beautiful, pleasing, sparkling eyes, giggling, and a powerful experience. Significantly, these positive effects extended to residents who had been exhibiting disruptive, loud behaviour as they became more relaxed, contented, and settled for a period of time.

*At the end of the activity I [noticed] that [this resident's] manner [had] changed from the interaction with George from one of vagueness and a far-away look to having energy in his approach and showing calmness and love. [Participant 3 log entry]*

*I heard [the resident] yelling out, “Help, Help, Help!” As soon as he saw me he stopped yelling and beckoned for me to come to him with George. He spent the rest of the time… touching and observing [the] movements of George. After leaving, I listened and he did not commence his usual yelling for help. Also I returned 20 minutes later to listen for yelling and did not hear [the resident] verbalizing.” [Participant 3 log entry]*

In addition to emotional and behavioural benefits, the participants described social benefits to the use of Paro. While there was clearly a positive impact from the use of Paro directly, some of the indirect benefits noted by participants included a deepening of relationships between therapists and the residents.

*There was one lady…she's a loner and she stays in her room at all times. Having…Sally placed in her arms, it just reminded her of her baby. She just opened up and her face just lit up…it was a just powerful moment and…it was just beautiful just to be there to experience it… I believe that she changed there and then on the spot. So even today, even though we've got a good rapport, I think it's even closer…. [Participant 3]*

One therapist used Paro to foster social connection by encouraging a group of residents to work together to create lyrics about Paro to a familiar old tune.

*They were all excited. It was a noisy atmosphere and everyone was talking at the same time, laughing, giggling and elbowing each other trying to come up some rhyming words. Some residents [came] up with funny words and some [were] not suitable, but it sounded very funny and they all laughed… Residents were using their brains it stimulation them. It was also good social interaction and a good laugh. Until next time, I am planning to record the song on CD when completed. [It's a] good sense of achievement for the residents. [Participant 2 log entry]*

Social and relationship benefits were noted to extend from other residents and carers to the family members, in one example prompting recall of memories.

*Both mother and daughter had a good laugh at the way her mother was talking to George; [the] daughter [said] “it reminds me [of] when I was little, she spoke to us like that, it's funny it makes me laugh”. [Participant 2 log entry]*

The participants in this study also observed that family members recognised the value of Paro and appreciated the positive impact that the robotic seals had for their loved one. In one case, the daughter of a resident, concerned that her mother would be distressed that she could not make her regular visit, specifically asked a participant that her mother be given some time with Paro.

*I took George this morning to see a resident, her daughter came to me the other day and said… “I won't be visiting mum … on Thursday, could you please take George or Sally up to her?” [Participant 1]*

### 3.3. Keeping the Momentum

This final theme reflects the participants' initial sense of responsibility towards ensuring the success of Paro as a therapeutic tool. Despite indicating that they had come into the trial project without any preconceived ideas, participants appeared hopeful rather than sceptical about the use of Paro. This attitude likely helped them to wholly embrace the possibilities of Paro. Therapists incorporated the robotic seals into their daily activities with residents and sought to educate and reduce scepticism amongst others as part of their role.

*But you do that… as an educator – I suppose that's part of me inside is making sure that people understand why we do it and we need to continue to keep the momentum happening. [Participant 3]*

Therapists were keen to share the benefits of Paro and in particular to* “include the nurses as well.”* They encouraged cynical staff members to observe Paro being used, understanding the power of witnessing the change in residents' behaviour.

*They came in and they observed him and they couldn't believe what he was doing, like singing all these songs - they've never ever heard this cranky man in their life sing, smile so much. They've never seen his teeth and here he is smiling and his face is lit up… Watch, observe and I said, he's your client, now you know what he can do… when he's cranky, you now can put a smile on his face. So that… Paro made him come out of his shell [and made] the staff more aware of what was actually happening…. [Participant 3]*

The advocacy for the use of Paro shown by the participants in this study was likely related to their own experiences of witnessing the benefits, which included a seemingly vicarious sharing of residents' emotions.

*It's just the reaction… They're in the moment on the spot… it just gives you joy, you can see their faces. [Participant 2]*

*I went and had a bit of a cry…. [Participant 3]*

These participants clearly saw the enduring value of Paro beyond the initial trial period. Their suggestions comprised using Paro to assist with care planning, activities of daily living (ADLs), and wound care and to reduce incidences of challenging behaviour. The consensus appeared to be that use of Paro required regularity and momentum to effect maximum benefits.

*I'd like to see them used daily, and different areas. If money wasn't an issue, or staffing wasn't an issue, it'd be lovely to be able to have them in each area. [Participant 1]*

## 4. Discussion

The findings of this study present an interesting overview of the impact of Paro on residents in an aged care facility from the perspective of the therapists who employed these robotic seals in their practice. These findings add to the existing contemporary literature about robot seals and their use with the elderly. Furthermore, this study expands knowledge from a qualitative perspective as well as in respect of what is known about the use of robot seals with the elderly beyond that of dementia care. These participants reported mixed reactions from residents, family, and staff. Giusti and Marti [27] suggest that a number of factors determine how an individual may interact with Paro, including the context and their personal history. Recognition of these factors, combined with the findings evident in this study, highlights the importance of care planning that respects and accommodates individual differences, preferences, and needs.

The potential impact of personal history explains the use of a seal form of the robot. While participants reported confusion for residents in some instances, the use of an animal with which the residents are unlikely to be familiar removes the potential impact of any existing preconceptions or experiences. A baby harp seal is an endearing and relatively innocuous creature to which an individual can direct their affection [19]. Physical and cognitive status are further factors that influence the reaction of those who come into contact with the robot and this can be seen from the findings described in this paper. In particular, participants noted that residents with dementia were likely to perceive Paro as a living thing, reflecting the results of Giusti and Marti [27].

The findings in this paper indicate that participants perceived a degree of negativity amongst some residents, their relatives, and other staff. This finding is in contrast with the work of both Heerink et al. [15] and Robinson et al. [11], who found that Paro was received favourably by these groups. Lack of understanding of the value and capability of the robotic seal no doubt contributed to the negative reaction from some who came into contact with it. Indeed, members of the research team themselves were initially unsure of the value or significance of these therapeutic tools when they were first encountered. As was also found by Bemelmans [26], sceptics of Paro can become enthusiasts once the positive effects are seen.

Some of the negativity towards the Paro might result from a perception that the use of robots with cognitively impaired individuals is humiliating and demeaning. This might particularly be the case when those who witness the resident interacting with the robotic seal lack an understanding of its therapeutic capability, such as untrained carers [14]. While recent work has discussed the ethical implications of using seals in the care of the elderly [13, 14, 19], no such specific concerns were evident in this study. Nonetheless, these findings highlight the importance of education for those people who work directly and indirectly with Paro to avoid ethical issues arising.

Past research identified therapeutic benefits emotionally [22, 26], physically [9], behaviourally [28], and socially [9, 21, 23, 24]. The research reported in this paper adds support to these findings for the use of Paro in improving mood, reducing challenging behaviour, and facilitating social interactions. As a result of its qualitative design, the current study also brought to light some of the processes involved in these therapeutic improvements in specific contexts. Participants reported positive responses to Paro that involved physical touch, verbal communication, and expressed affection, similar to those observed between pet owners and their pets [29]. It is hypothesised that such benefits could improve the quality of life and reduce the distress of residents. By doing so, they have the potential to reduce the worry and distress of family members and the stress and potential for burnout in residential staff.

Reduced loneliness has been identified previously as a benefit of Paro [9, 23] and the observations of participants in this study suggested two main processes in this respect: the establishment of a direct relationship with the robotic seal and its use in facilitating better social connections with others. Isolated and withdrawn residents talked to Paro in a conversational manner and end-of-life residents verbalised their internal world, in both contexts seemingly reducing a sense of being alone. This finding highlights distinctions between the loss of ability to communicate, the loss of desire to communicate, and the loss of opportunities to communicate.

Chang et al. [22] described Paro as a “*good social mediator*” (p. 102) and participants in this study provided descriptive examples of such processes occurring between groups of residents, residents and staff, and residents and family members. These findings suggest that staff members can use the robotic seal as a rapport-building tool with otherwise socially and verbally withdrawn residents. In groups, it can be used as an external and nonthreatening focal point for group activities. With family members, Paro can be used for both day-to-day conversation and reminiscence, in line with Sung et al. [28].

Calo et al. [19] have previously written about the ethics and value of replacing human relationships with robot relationships and the issue of using a robotic seal as a replacement social relationship was raised in this research. It was beyond the scope of this research to consider whether the relationship between human and Paro is best understood as an attachment relationship as occurs with pets [30] or where Paro is a transitional object as proposed by Taggart et al. [31].

It was clear to the research team that the participants in this study were very excited at the results of their work with the residents and Paro. They expressed a desire to teach others about the benefits of using Paro in the hope of motivating them to employ it as a therapeutic tool. Beyond the social and behavioural benefits described by the participants, there is clear potential for Paro to be used effectively in assisting residents with activities of daily living such as at meal times, in preparation for showering, and at night when they are unable to sleep. Although Bemelmans et al. [26] found no significant benefit of using Paro in care activities, that finding was confounded by the limited number of care activities examined and an associate learning curve in the use of the robot for this purpose. Those authors did, however, find that Paro had a significant therapeutic impact. Previous work has shown that Paro can alter the psychosocial environment [25] and function as a social catalyst [24] thereby establishing a context where elderly residents are more likely to be receptive to care interventions. The findings of the study described in this paper confirm the positive impact of Paro detailed in early work and suggest that there is even greater scope for its use in the support of residents in aged care facilities.

### 4.1. Recommendations and Limitations

A number of recommendations arise from this study. In respect of practice, it is clear that if robotic seals are to be introduced into the practice environment, it is necessary for those who will be involved in their use to be adequately prepared. The gradual introduction of Paro into the clinical or residential setting, with increasing visibility and accessibility, may maximise the potential for success. Sharkey [14] believes that therapeutic benefits of robots are the result of skilled use. Thus, the carer must be adequately trained before they are deployed in any setting. Given the positive effect of Paro suggested by this and earlier work, the expansion of its use to include other caregivers, in particular nurses, may broaden its potential therapeutic reach.

Future research is recommended that validates the findings of this study and explores issues that arise from this work. Perspectives of other caregivers, particularly those who express negativity towards the robotic seal, may identify barriers to its use. Studies on the value of Paro in improving caregiver wellbeing and reducing staff burnout are also warranted. Longitudinal work would also confirm the long-term therapeutic value of Paro found by Jøranson et al. [25] and support Sabanovic et al.'s [23] assertion that the benefits are not simply due to a novelty effect. It is acknowledged that the positive impact observed by the participants may be attributable in part to their presence and not confined to the therapeutic value of Paro, consistent with the assertions of Jøranson et al. [25]. A comparative trial using standard therapeutic measures as an intervention, such as what was used by those authors, may highlight the unique value of Paro in the provision of care.

The major limitation of this study relates to the small sample size and its focus on a single facility. Nonetheless, the findings reinforce those of earlier studies and provide greater understanding of the potential value of Paro and other social responsive robots to those who work in facilities such as the one described in this paper.

## 5. Conclusions

Ensuring a safe, therapeutic, and respectful environment for the elderly members of our society is a key concern for people working in residential aged care facilities. Both professional caregivers and significant others work with limited resources in managing the often complex physical, cognitive, social, and emotional needs of the elderly. Any and all tools that can aid in promoting the wellbeing of aged residents should not be dismissed until potential has been fully assessed. This paper has presented the findings of a study into the use of the Paro robotic seal by recreational therapists in an aged care facility. While it is clear that not all those who come in contact with these robotic devices will see their value, there is evidence that these tools can have a positive impact that warrants further exploration. The potential benefits clearly justify the investment.
